# ATR-FTIR Analysis of Orthodontic Invisalign^®^ Aligners Subjected to Various In Vitro Aging Treatments

**DOI:** 10.3390/ma14040818

**Published:** 2021-02-09

**Authors:** Lucia Memè, Valentina Notarstefano, Francesco Sampalmieri, Giulia Orilisi, Vincenzo Quinzi

**Affiliations:** 1 Department of Clinical Sciences and Stomatology, Polytechnic University of Marche, Via Tronto 10, 60126 Ancona, Italy; lucia.meme@hotmail.it (L.M.); f.sampalmieri@univpm.it (F.S.); g.orilisi@pm.univpm.it (G.O.); 2 Department of Life and Environmental Sciences, Polytechnic University of Marche, Via Brecce Bianche, 60131 Ancona, Italy; v.notarstefano@univpm.it; 3 Department of Life, Health & Environmental Sciences, Postgraduate School of Orthodontics, University of L’Aquila, P.le Salvatore Tommasi 1, Ed. Delta 6, 67100 L’Aquila, Italy

**Keywords:** orthodontic aligners, polyurethane, polyester, aging treatments, ATR-FTIR spectroscopy

## Abstract

Clear and removable tooth aligners for orthodontics treatments have become an increasingly popular alternative to fixed appliances. Even if protocols suggest removing aligners before eating or drinking, most patients retain them when they drink beverages. Alterations in the material during the daily use could determine a reduction in the application forces, affecting the desired orthodontic movement; the knowledge of how this material reacts when subjected to different aging processes is mandatory to establish the predictability of the orthodontic treatment. According to this, the aim of the present study was to assess a new objective approach, coupling spectroscopic and chemometric tools, to evaluate the changes occurring in Invisalign^®^ aligners, the most widely used brand, exposed in vitro to coffee, tea, Coca Cola^®^ and UV radiation for 24 and 48 h. In particular, ATR-FTIR spectroscopy was utilized to characterize, at the molecular level, the chemical and color modifications in the surfaces of the appliances; the obtained data were submitted to PCA and one-way ANOVA and Tukey’s multiple comparison test. Moreover, a colorimetry analysis was carried out to evaluate any changes in color and transparency. Coffee and tea samples displayed the major color changes between the tested groups. The differences highlighted in the spectral features of coffee, tea and UV-treated samples were mainly ascribable to color and transparency changes, because the chemical properties remained unaltered.

## 1. Introduction

The development of CAD/CAM technology has permitted the use of thermoplastic materials to fabricate clear and removable tooth aligners for orthodontics treatments, which have become an increasingly popular alternative treatment to fixed appliances [1,2]. With respect to traditional orthodontic treatment modalities, it offers various advantages, such as less patient discomfort, better aesthetics, easier periodontal maintenance, and less chair time for dentists [3,4,5]. The first report regarding the use of a flexible aligners for orthodontic treatment dates back to 1945 [6]. Subsequently, various types of invisible retainers have been developed. To date, there are more than 27 different clear aligner brands available in the global market: Invisalign^®^ is the most widely used brand, with over five million patients worldwide [7].

Polyurethane, a versatile engineering thermoplastic polymer with excellent physical properties, chemical and abrasion resistance and ease of process, is the first material that was used for clear aligners [8]. Over time, it has been proven that polyurethane is not an inert material; it is affected by heat, moisture, and prolonged contact with salivary enzymes [9].

In 2001, Invisalign^®^ (Align Technology, San Jose, CA, USA) started to produce aligners made with a material called Proceed30 (PC30), a polymer mixture that unfortunately did not meet all the physical-chemical and clinical requirements for orthodontic tooth movement [10,11]. Hence, in 2013, this material was substituted with Exceed30 (EX30), composed of polyurethane methylene diphenyl diisocyanate 1,6-hexanediol. EX30 showed better properties with respect to PC30; indeed, it exhibited an elasticity 1.5-fold greater than that of PC30, facilitating the removal and insertion of the aligners. In addition, the aligners were four-fold more adaptable than that produced with PC30 [12].

Around 70–80% of orthodontic treatments require midcourse correction, case refinement or conversion to fixed appliances before the end of treatment [13,14]; therefore, in 2013, a new innovative polymer called SmartTrack^®^ (LD30) was introduced. It is a multilayer aromatic thermoplastic polyurethane/copolyester polymer covered by patent protection. LD30 has three main advantages compared with EX30: greater consistency of application of orthodontic forces, greater elasticity, chemical stability, and an even more precise and comfortable aligner fit [15,16].

The clinical protocol applied to removable appliances establishes the intraoral placement of the aligners for a maximum of one or two weeks, after which the aligner must be replaced with the sequential ones. Patients are normally asked to remove aligners every time they consume solid and liquid foods, except water, and when they brush or floss their teeth [17]. However, most patients retains aligners when they drink beverages, or when they smoke. This habit could lead to staining of the aligners and, as a further extent, to chemical changes in their composition [18]. The knowledge of how this material changes its chemical composition when subjected to different aging processes is an important tool for establishing the predictability of the orthodontic treatment. In fact, the alteration of the material during daily use could determine a reduction in the application forces and affect the desired orthodontic movement. There are only few studies that have examined the color stability of a limited set of brands available on the market, subjected to staining agents [16,18,19]. In 2020, Bernard et al. evaluated the color stability of the polymer composing three different American brands of aligners. They concluded that the Invisalign^®^ aligners were more prone to pigmentation after a 12 h or a seven-day exposure to coffee or red wine compared to the ClearCorrect^®^ or Minor Tooth Movement^®^ devices [15]. Finally, it is reported in the literature that aromatic polyurethane undergoes photodegradation with gradual yellowing, coupled, in some cases, with changes in physical, chemical, and mechanical properties when subjected to UV radiation [20,21,22,23,24].

In this light, the present study aimed to increase the knowledge on the response of Invisalign^®^ aligners, the most widely applied brand, to different aging treatments, providing a new objective approach coupling ATR-FTIR spectroscopy with multivariate and univariate analyses, able to evaluate the chemical and color modifications occurring in the polymeric matrix. To this purpose, different portions of aligners were exposed in vitro for 24 and 48 h at 37 °C to coffee, tea, and Coca Cola^®^ solutions, and, as further extent, to UV radiation (254 nm); time points were intentionally selected in order to stress the material. Samples were analyzed by ATR-FTIR spectroscopy, and spectral data were subjected to principal component analysis. Specific band height ratios were calculated and statistically analyzed. Samples were also investigated by colorimetry analysis.

## 2. Materials and Methods

### 2.1. Experimental Design and Aging Treatments

The study was performed on 12 new Invisalign^®^ aligners. From each aligner, 2 portions (ca. 0.5 × 0.5 cm^2^) were cut in the lower sixth molar area, for a total of 24 pieces, which were divided into 4 experimental groups (*n* = 6 for each experimental group). Aligner portions were immersed in the following solutions: (1) sugar-free long coffee (Lavazza^®^) (CF samples); (2) sugar-free tea (Earl Grey Twinings, 30 mg/150 mL) (T samples); and (3) Coca Cola^®^ (Coca-Cola^®^ Company, Atlanta, GA, USA) (CC samples). Moreover, another aliquot was irradiated with UV radiation at 254 nm (UV samples). During all the treatments, staining solutions were maintained at 37 °C, in order to simulate the physiological oral environment, and were stirred, to allow a uniform contact with samples. Before IR measurements, samples were washed with milliQ water and then dried with a stream of nitrogen.

### 2.2. ATR-FTIR Measurements

The infrared analysis of the aligners was carried out with a Bruker Invenio FTIR spectrometer equipped with a Platinum ATR accessory for reflectance measurements (OPUS 7.5 software package, Bruker Optics, Ettlingen, Germany).

The ATR-FTIR spectra were collected at room temperature on dried samples just before the in vitro aging treatments (considered as a control group, Ctrl) and at the time points of 24 and 48 h. Five spectra were acquired in reflection mode in the spectral range 4000–600 cm^−1^ (spectral resolution 4 cm^−1^, 64 scans) on the external surface of each sample. A background spectrum was collected on the clean diamond crystal, before each sample measurement. All IR spectra were converted into Absorbance mode, interpolated in the spectral range 1800–700 cm^−1^, and two-points baseline linear fitted (OPUS 7.5 software, Bruker Optics). These preprocessed spectra were then subjected to principal component analysis (PCA; OriginPro 2018b software; OriginLab Corporation, Northampton, MA, USA) [25].

For each experimental group, height values of specific bands were calculated by using the Integration routine (K mode) (OPUS 7.5 software package, Bruker Optics, Ettlingen, Germany).

### 2.3. Colorimetry Analysis

Color changes due to aging treatments were determined by using an automatic reflectance colorimeter (Konica-Minolta Chroma-Meters CR-400, Tokyo, Japan). On each sample, three measurements were performed, after 24 and 48 h of treatment. All the samples were washed with milliQ water and then dried with a stream of nitrogen before starting the measurements. For each sample, the total color change (∆E*) was calculated according to Daniele et al. [26]. Data were converted into the National Bureau of Standards (NBS) system by using the equation NBS = ∆E* × 0.92, in order to offer a clinical interpretation (Table 1).

### 2.4. Statistical Analysis

Normally distributed data deriving from ATR-FTIR and colorimetry analyses were presented as mean ± standard deviation (S.D.). Significant differences between experimental groups were determined by means of factorial analysis of variance (one-way ANOVA), followed by Tukey’s multiple comparison test, using the statistical software package Prism6 (GraphPad Software, Inc., San Diego, CA, USA). Significance was set at *p* < 0.05.

## 3. Results

New Invisalign^®^ aligners were subjected for 24 and 48 h in vitro to different aging treatments, including three commonly consumed drinks (coffee (CF), tea (T) and Coca Cola^®^ (CC)), and, as further extent, to UV radiation (UV, λ = 254 nm). Samples were analyzed by attenuated total reflectance–Fourier-transform infrared spectroscopy (ATR-FTIR) to assess possible changes in the molecular composition and physical profile of the polymeric matrix.

**Figure 1:**
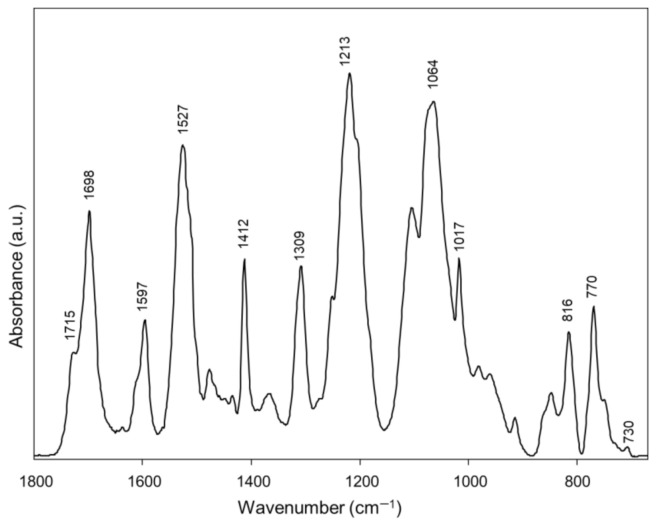
IR spectrum of a Ctrl portion of Invisalign^®^ aligner.

The average IR spectrum of Ctrl Invisalign^®^ aligners is reported in Figure 1. The following peaks have been identified: 1715 cm^−1^ and 1698 cm^−1^ (stretching mode of the C=O groups, free and H-bonded, respectively); 1597 cm^−1^ (aromatic rings vibrations); 1527 cm^−1^ (N–C=O moiety vibrations); 1412 cm^−1^ (bending modes of C–H bonds); 1309 cm^−1^ (C=O vibrations); 1213 cm^−1^ (stretching modes of C–N bonds); 1064 cm^−1^ and 1017 cm^−1^ (stretching modes of C–O–C bonds); 816 cm^−1^, 770 cm^−1^ and 730 cm^−1^ (bending modes of C–H bonds in aromatic rings) [18,26,27,28]. The comparison of this average IR spectrum with the spectral library of the KnowItAll software (Wiley Sciences Solutions, John Wiley & Sons, Inc., Hoboken, NJ, USA) fitted with a copolymer formed by aromatic polyurethanes and polyesters (Hit Quality Index, HQI > 85.0).

**Figure 2:**
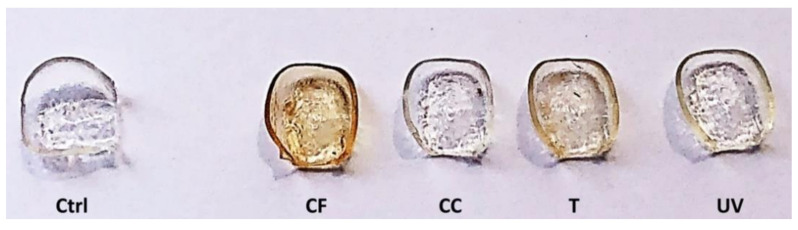
Invisalign^®^ aligners portions after 48 h of treatments with coffee (CF), Coca Cola^®^ (CC), tea (T) and UV radiation (UV), compared to the control (Ctrl).

The visual inspection of aligners after 48 h of in vitro aging treatments evidenced a marked darkening in CF and T samples, with respect to the Ctrl samples; only a mild yellowing was observed in the UV samples, while no color alteration was highlighted in CC aligners (Figure 2).

These results were confirmed and quantified by the colorimetry analysis, performed according to the NBS system (Table 1). The colorimeter values of Ctrl, reported according to the Commission Internationale de l’Eclairage L*a*b* color system (CIE L*a*b*) [26], were: 70.99 (L*), −0.06 (a*), 1.77 (b*). The means and standard deviations of the ΔE* (the color change) values are presented in Table 2.

**Figure 3:**
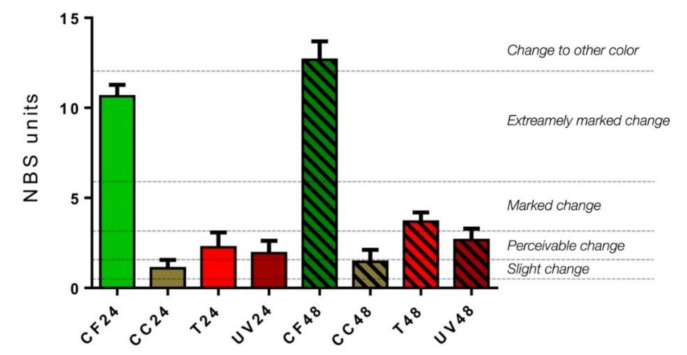
Colorimetric measurements (expressed by NBS system) of aligners subjected to the following aging treatment at 24 and 48 h: coffee (CF24 and CF48), Coca Cola^®^ (CC24 and CC48), tea (T24 and T48) and UV radiation treatments (UV24 and UV48).

According to the NBS system (Figure 3), CF samples showed an extremely marked change, more pronounced at 48 h than 24 h (CF24, NBS = 10.64 ± 0.64; CF48, NBS = 12.67 ± 1.03; *p* < 0.05). CC samples displayed slight changes both at 24 and 48 h (CC24, NBS = 1.09 ± 0.47; CC48, NBS = 1.45 ± 0.67; *p* > 0.05). With regard to T samples, a perceivable change was detected at 24 h, which became more marked at 48 h (T24, NBS = 2.26 ± 0.82; T48, NBS = 3.68 ± 0.52; *p* < 0.05). Finally, UV samples showed a perceivable change both at 24 and 48 h (UV24, NBS = 1.93 ± 0.69; 2.65 ±0.64; *p* < 0.05).

**Figure 4:**
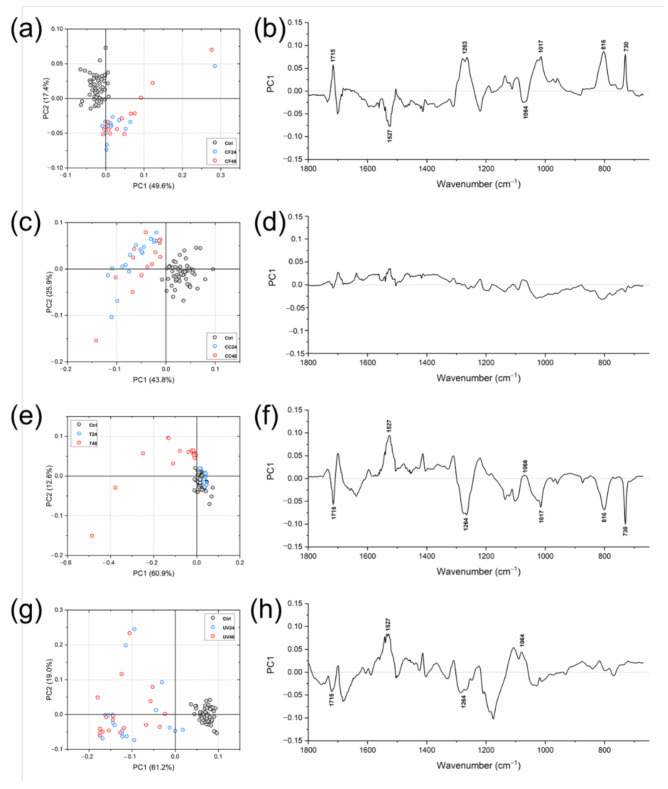
PCA score plots of: (**a**) Ctrl/CF24/CF48; (**c**) Ctrl/CC24/CC48; (**e**) Ctrl/T24/T48, and (**g**) Ctrl/UV24/UV48. PC1 loading spectra of: (**b**) Ctrl/CF24/CF48; (**d**) Ctrl/CC24/CC48; (**f**) Ctrl/T24/T48, and (**h**) Ctrl/UV24/UV48.

To evaluate the eventual changes caused the chemical composition of the aligners by in vitro aging treatments, the following IR spectral populations were subjected to multivariate analysis: Ctrl/CF24/CF48, Ctrl/CC24/CC48, Ctrl/T24/T48, and Ctrl/UV24/UV48 (Figure 4). From the PCA score plots, the following considerations could be drawn: (i) in all PCA scores plots, Ctrl spectra appeared well grouped in a compact cluster; (ii) regarding Ctrl/CF24/CF48 (Figure 4a), Ctrl/CC24/CC48 (Figure 4c) and Ctrl/UV24/UV48 (Figure 4g) PCA score plots, a satisfactory segregation along the PC1 axis was observed among Ctrl, CF, CC, and UV spectra (explained variances of 49.6%, 43.8%, and 61.2%, respectively); moreover, CF, CC and UV spectra appeared very scattered and no separation was found with relation with time (between 24 and 48 h); (iii) a different trend was observed in PCA score plot of Ctrl/T24/T48, with CTRL and T24 spectra superimposed in a single compact cluster—a good segregation was found with respect to T48 spectra (explained variance along PC1 axis 60.9%), which, however, appeared very scattered (Figure 4e). To evaluate the spectral features responsible for the separation in PCA score plots, PC1 loading spectra were also analyzed: (i) with respect to Ctrl samples, CF and T aligners showed spectral changes mainly in the free C=O bonds (1715 cm^−1^), N–C=O bonds (1527 cm^−1^), C–N bonds (1263 cm^−1^), C–O–C bonds (1064 cm^−1^ and 1017 cm^−1^) and C–H in the aromatic rings (816 cm^−1^ and 730 cm^−1^) (Figure 4b,f); (ii) regarding samples treated with Coca Cola^®^ (CC samples), no spectral modifications were found, either at 24 or 48 h (Figure 4d); (iii) irradiation with UV light caused changes in the free C=O bonds (1715 cm^−1^), N–C=O bonds (1527 cm^−1^), C–N bonds (1263 cm^−1^), and C–O–C bonds (1064 cm^−1^) (Figure 4h).

**Figure 5:**
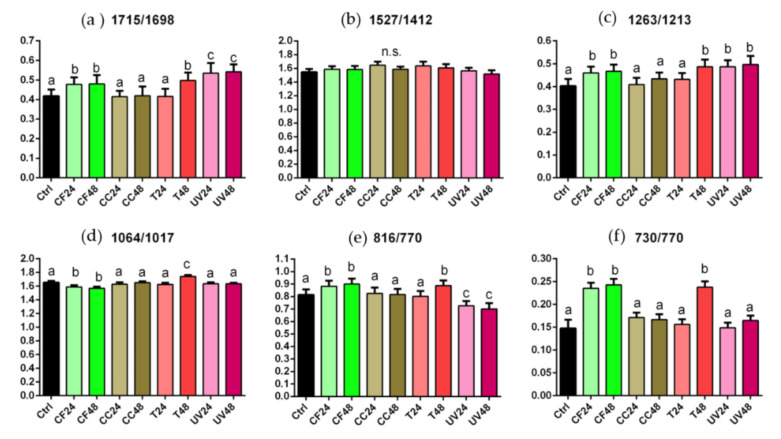
Statistical analysis of the following band height ratios calculated for Ctrl, CF24, CF48, CC24, CC48, T24, T48, UV24, and UV48: 1715/1698 (**a**), 1527/1412 (**b**), 1263/1213 (**c**), 1064/1017 (**d**), 816/770 (**e**) and 730/770 (**f**). Different letters indicate statistically significant differences among groups (*p* < 0.05; one-way ANOVA and Tukey’s multiple comparison test).

To evaluate the extent of these modifications in terms of chemical composition of the polymeric matrix, specific band height ratios were calculated and are reported in Figure 5. By their statistical analysis, the following considerations can be drawn: (i) the 1715/1698 and 1263/1213 ratios, describing the C=O and C-N bonds, free and H-bonded, respectively, displayed an analogous trend: in both cases, no statistically significant differences were found by comparing CC and T24 samples with respect to the Ctrl (*p* > 0.05), while statistically significant higher values were observed in CF, T48 and UV samples (*p* < 0.05); (ii) no statistically significant differences were displayed in the 1527/1412 ratio, representing the ratio between the amide linkage and C–H bonds (*p* > 0.05); (iii) regarding the 1064/1017 ratio, related to C–O–C moiety, no statistically significant differences were found in CC, T24 and UV samples with respect to the Ctrls (*p* > 0.05), while statistically significant changes were observed in CF and T48 samples (exhibiting the lowest and highest values, respectively) (*p* < 0.05); (iv) a similar trend was observed regarding the 816/770 and 730/770 ratios, both related to the vibrational modes of C–H bonds in the aromatic rings; in particular, no statistically significant differences were found by comparing CC and T24 samples with the Ctrls (*p* > 0.05), while, higher values were observed in CF and T48 samples (*p* < 0.05); UV samples showed values similar to the Ctrls regarding the 730/770 ratio (*p* > 0.05), while statistically significant lower values were displayed with respect to the 816/770 ratio (*p* < 0.05).

## 4. Discussion

Invisalign^®^ is the most widely employed brand of clear and removable tooth aligners for orthodontic treatments [7]. Its composition has been improved by the introduction of a new innovative polymer called SmartTrack^®^ (LD30), which exhibits high consistency of application of orthodontic forces, high elasticity, chemical stability, and a precise and comfortable aligner fit [15,16].

Some studies are reported in the literature, having performing colorimetry tests and FTIR spectroscopy on dental aligners; however, to the best of our knowledge, no multivariate and univariate analysis of IR data has been performed [16,18].

In this study, for the first time, ATR-FTIR spectroscopy was coupled with multivariate and univariate analyses to assess an objective analytical tool for quality control of clear tooth aligners. Color changes were also highlighted, following a standard protocol [26]. The chemical and color/transparency changes determined in the tested aligners, by in vitro exposure to commonly used beverages—Coffee, Coca Cola^®^, and tea—Were investigated. Given the known aggressive effects of UV radiation on polyurethane polymers, it was also included in the study, and its effects were compared with those of the tested beverages. Time points of 24 and 48 h were chosen in order to stress the material and, hence, to ensure that the possible molecular and color changes were detectable.

The color stability of orthodontic materials can be negatively influenced by staining beverages, causing aesthetic changes related to the loss of transparency. Our results are in agreement with those reported by Chen et al. and Bernard et al. [16,18]. Significant differences were highlighted mainly in coffee- and tea-treated samples at 48 h. Conversely, at the same time point, UV and Coca Cola^®^ treatments caused perceivable changes.

With regards to multivariate analysis, PCA was utilized to assess the presence of possible differences in the spectral features of aged aligners (PCA score plots). Moreover, by the analysis of PC1 loadings, the most discriminant spectral features were identified, from which specific band height ratios were calculated and statistically analyzed to evaluate the response of the material to the tested in vitro aging conditions. Ctrl spectra results were generally grouped in more compact clusters than the other treated groups, consistent with the homogeneous chemical profile of the polymeric matrix; conversely, spectra belonging to coffee, Coca Cola^®^ and UV groups showed a more scattered distribution, with no separation according to treatment time (24 and 48 h), suggesting an inhomogeneous response of the material to these aging treatments. In particular, regarding the coffee-treated samples, a good segregation of Ctrl spectra was observed, along PC1 and PC2 axes, with respect to both CF24 and CF48 ones, that were grouped in a single scattered cluster. This result is possibly ascribable to the major color changes observed in CF samples at both time points (*p* < 0.05). Conversely, a minor segregation was found among Ctrl spectra and both CC24 and CC48 results; moreover, a slight color change was detected in CC48. Regarding tea-treated samples, a good segregation of T48 spectra was observed, along the PC2 axis, compared to both Ctrl and T24 ones, which were grouped in a single compact cluster; moreover, a significant color change was detected mainly in T48 (*p* < 0.05). These findings suggested that tea treatment caused changes both in terms of color and chemical composition, although only after 48 h. Although UV24 and UV48 spectra were very scattered, they were clearly separated along the PC1 axis with respect to the Ctrls; a slight visible yellowing was observed mainly after 48 h of treatment.

According to these results, it is possible to hypothesize that the spectral differences highlighted in coffee and tea samples are mostly ascribable to changes in color rather than chemical composition. Accordingly, Bradley and colleagues reported that no relevant chemical difference was found in Invisalign^®^ appliances after intraoral aging, but only changes in the mechanical properties [23]. Moreover, in 2016, Liu and colleagues analyzed the color stability in different aligners, including Invisalign^®^, finding that the polymer-based structure of aligners did not exhibit significant chemical differences before and after the immersions in three staining solutions (coffee, black tea, and red wine) [16].

One study analyzed the physiochemical and mechanical characterization of orthodontic invisible aligners. The authors concluded that the immersion in red wine and coffee solutions gave the highest color variations, while nicotine and artificial saliva showed negligible changes [26]. Our findings are in agreement with this study, because the coffee- and tea-treated aligners showed the most representative color changes among all the tested groups. This could be attributed to the chemical composition of the aligners, which contained polar groups –NHCOO– that easily create hydrogen links interacting with the hydrophilic groups of the pigments, thus facilitating their absorption into the material [16,29]. Hence, tea and coffee seemed to bind more to the material, slightly modifying the chemical composition (1715/1698, 1263/1213 and 1064/1017 band height ratios, *p* < 0.05).

This was an in vitro study; therefore, it does not replicate the normal oral conditions where individuals wear the appliances during the recommended time. Even if it has been concluded that the mechanical properties, surface molecular structure, and internal structure of Invisalign^®^ LD30 material were not significantly affected by the oral environment [30], further investigations should be performed to evaluate the effects of artificial saliva on the chemical and color modifications of the aligners.

According to the obtained results, clinicians should instruct patient to not drink coffee and tea wearing aligners, both for aesthetic reasons, because the color change is unacceptable in the context of worn aligners, and because the chemical composition of the material could be slightly modified, influencing the planned dental movements. Further investigations will be performed, also exploiting Raman microspectroscopy, on a wider variety of brands of clear aligners, to compare their response, in terms of chemical and color modifications, to different aging and temperature conditions, and, hence, to better depict the optimal conditions in which they can be employed.

## 5. Conclusions

In this in vitro study, for the first time, the coupling of ATR-FTIR spectroscopy with multivariate and univariate analysis, enabled the defining of specific IR band height ratios to evaluate the chemical and color changes caused by different aging treatments in Invisalign^®^ aligners.

Altogether, the obtained results suggest that the chemical properties of the analyzed aligners are not affected by the exposure to the tested beverages. The chemical characteristics were mostly preserved after all treatments, suggesting that the mechanical properties were also conserved; only coffee and tea caused significant color changes, due to the attack of their pigments on the polymer. To avoid this aesthetical inconvenience, clinicians should suggest that patients prevent the contact of aligners with staining agents such as coffee and tea.

## Figures and Tables

**Table 1:** National Bureau of Standard ratings.

National Bureau of Standards Units	Descriptions of Color Changes
0.0–0.5	Trace: Extremely slight change
0.5–1.5	Slight: Slight change
1.5–3.0	Noticeable: Perceivable
3.0–6.0	Appreciable: Marked change
6.0–12.0	Much: Extremely marked change
12.0 or more	Very much: Change to another color

**Table 2:** Comparisons of color change (ΔE*) values of aligners subjected to different aging treatments at 24 and 48 h. (CF, coffee; CC, Coca Cola^®^; T, tea; UV, UV radiation). Different uppercase (a, b, c, d, and e) letters indicate statistically significant differences among groups (*p* < 0.05; one-way ANOVA and Tukey’s multiple comparison test).

Aging Treatments	24 h	48 h
CF	11.57 (±0.69) ^a^	13.78 (±1.12) ^d^
CC	1.19 (±0.51) ^b^	1.58 (±0.69) ^b^
T	2.46 (±0.89) ^c^	4.00 (±0.57) ^e^
UV	2.10 (±0.75) ^b,c^	2.88 (±0.69) ^c,e^

## Data Availability

The data presented in this study are available within the present article.

## References

[1] Rosvall M.D., Fields H.W., Ziuchkovski J., Rosenstiel S.F., Johnston W.M. (2009). Attractiveness, Acceptability, and Value of Orthodontic Appliances. Am. J. Orthod. Dentofac. Orthop..

[2] Zheng M., Liu R., Ni Z., Yu Z. (2017). Efficiency, Effectiveness and Treatment Stability of Clear Aligners: A Systematic Review and Meta-Analysis. Orthod. Craniofac. Res..

[3] Rossini G., Parrini S., Castroflorio T., Deregibus A., Debernardi C.L. (2015). Efficacy of Clear Aligners in Controlling Orthodontic Tooth Movement: A Systematic Review. Angle Orthod..

[4] Buschang P.H., Shaw S.G., Ross M., Crosby D., Campbell P.M. (2013). Comparative Time Efficiency of Aligner Therapy and Conventional Edgewise Braces. Angle Orthod..

[5] Fujiyama K., Honjo T., Suzuki M., Matsuoka S., Deguchi T. (2014). Analysis of Pain Level in Cases Treated with Invisalign Aligner: Comparison with Fixed Edgewise Appliance Therapy. Prog. Orthod..

[6] Kesling H.D. (1945). The Philosophy of the Tooth Positioning Appliance. Am. J. Orthod. Oral Surg..

[7] Weir T. (2017). Clear Aligners in Orthodontic Treatment. Aust. Dent. J..

[8] Lu Q.-W., Macosko C.W. (2004). Comparing the Compatibility of Various Functionalized Polypropylenes with Thermoplastic Polyurethane (TPU). Polymer.

[9] Schuster S., Eliades G., Zinelis S., Eliades T., Bradley T.G. (2004). Structural Conformation and Leaching from in Vitro Aged and Retrieved Invisalign Appliances. Am. J. Orthod. Dentofac. Orthop..

[10] Lagravère M.O., Flores-Mir C. (2005). The Treatment Effects of Invisalign Orthodontic Aligners: A Systematic Review. J. Am. Dent. Assoc..

[11] Phan X., Ling P.H. (2007). Clinical Limitations of Invisalign. J. Can. Dent. Assoc..

[12] Condo’ R., Pazzini L., Cerroni L., Pasquantonio G., Lagana’ G., Pecora A., Mussi V., Rinaldi A., Mecheri B., Licoccia S. (2018). Mechanical Properties of “Two Generations” of Teeth Aligners: Change Analysis during Oral Permanence. Dent. Mater. J..

[13] Djeu G., Shelton C., Maganzini A. (2005). Outcome Assessment of Invisalign and Traditional Orthodontic Treatment Compared with the American Board of Orthodontics Objective Grading System. Am. J. Orthod. Dentofac. Orthop..

[14] Kuncio D., Maganzini A., Shelton C., Freeman K. (2007). Invisalign and Traditional Orthodontic Treatment Postretention Outcomes Compared Using the American Board of Orthodontics Objective Grading System. Angle Orthod..

[15] Bräscher A.-K., Zuran D., Feldmann R.E., Benrath J. (2016). Patient Survey on Invisalign® Treatment Comparing [Corrected] the SmartTrack® Material to the Previously Used [Corrected] Aligner Material. J. Orofac. Orthop..

[16] Liu C.-L., Sun W.-T., Liao W., Lu W.-X., Li Q.-W., Jeong Y., Liu J., Zhao Z.-H. (2016). Colour Stabilities of Three Types of Orthodontic Clear Aligners Exposed to Staining Agents. Int. J. Oral Sci..

[17] Gracco A., Mazzoli A., Favoni O., Conti C., Ferraris P., Tosi G., Guarneri M.P. (2009). Short-Term Chemical and Physical Changes in Invisalign Appliances. Aust. Orthod. J..

[18] Bernard G., Rompré P., Tavares J., Montpetit A. (2020). Colorimetric and Spectrophotometric Measurements of Orthodontic Thermoplastic Aligners Exposed to Various Staining Sources and Cleaning Methods. Head Face Med..

[19] Lombardo L., Arreghini A., Maccarrone R., Bianchi A., Scalia S., Siciliani G. (2015). Optical Properties of Orthodontic Aligners—Spectrophotometry Analysis of Three Types before and after Aging. Prog. Orthod..

[20] Rosu D., Rosu L., Cascaval C.N. (2009). IR-Change and Yellowing of Polyurethane as a Result of UV Irradiation. Polym. Degrad. Stab..

[21] Wang H., Wang Y., Liu D., Sun Z., Wang H. Effects of Additives on Weather-Resistance Properties of Polyurethane Films Exposed to Ultraviolet Radiation and Ozone Atmosphere. https://www.hindawi.com/journals/jnm/2014/487343/.

[22] Papadopoulou A.K., Cantele A., Polychronis G., Zinelis S., Eliades T. (2019). Changes in Roughness and Mechanical Properties of Invisalign® Appliances after One- and Two-Weeks Use. Materials.

[23] Bradley T.G., Teske L., Eliades G., Zinelis S., Eliades T. (2016). Do the Mechanical and Chemical Properties of InvisalignTM Appliances Change after Use? A Retrieval Analysis. Eur. J. Orthod..

[24] Jaggy F., Zinelis S., Polychronis G., Patcas R., Schätzle M., Eliades G., Eliades T. (2020). ATR-FTIR Analysis and One-Week Stress Relaxation of Four Orthodontic Aligner Materials. Materials.

[25] Notarstefano V., Gioacchini G., Giorgini E., Montik N., Ciavattini A., Polidori A.R., Candela F.A., Vaccari L., Cignitti M., Carnevali O. (2020). The Impact of Controlled Ovarian Stimulation Hormones on the Metabolic State and Endocannabinoid System of Human Cumulus Cells. Int. J. Mol. Sci..

[26] Daniele V., Macera L., Taglieri G., Di Giambattista A., Spagnoli G., Massaria A., Messori M., Quagliarini E., Chiappini G., Campanella V. (2020). Thermoplastic Disks Used for Commercial Orthodontic Aligners: Complete Physicochemical and Mechanical Characterization. Materials.

[27] Bandekar J., Klima S. (1991). FT-IR Spectroscopic Studies of Polyurethanes Part I. Bonding between Urethane COC Groups and the NH Groups. J. Mol. Struct..

[28] Characterization of Polyurethane Resins by FTIR, TGA, and XRD-Trovati-2010-Journal of Applied Polymer Science-Wiley Online Library. https://onlinelibrary.wiley.com/doi/full/10.1002/app.31096.

[29] Fernandes A.B.N., Ruellas A.C.O., Araújo M.V.A., Sant’Anna E.F., Elias C.N. (2014). Assessment of Exogenous Pigmentation in Colourless Elastic Ligatures. J. Orthod..

[30] Fang D., Li F., Zhang Y., Bai Y., Wu B.M. (2020). Changes in Mechanical Properties, Surface Morphology, Structure, and Composition of Invisalign Material in the Oral Environment. Am. J. Orthod. Dentofac. Orthop..

